# Emerging evidence implicating a role for neurexins in neurodegenerative and neuropsychiatric disorders

**DOI:** 10.1098/rsob.210091

**Published:** 2021-10-06

**Authors:** Katelyn Cuttler, Maryam Hassan, Jonathan Carr, Ruben Cloete, Soraya Bardien

**Affiliations:** ^1^ Division of Molecular Biology and Human Genetics, Department of Biomedical Sciences, Stellenbosch University, Cape Town, South Africa; ^2^ Division of Neurology, Department of Medicine, Faculty of Medicine and Health Sciences, Stellenbosch University, Cape Town, South Africa; ^3^ South African Medical Research Council Bioinformatics Unit, South African National Bioinformatics Institute, University of the Western Cape, Cape Town, South Africa; ^4^ South African Medical Research Council/Stellenbosch University Genomics of Brain Disorders Research Unit, Cape Town, South Africa

**Keywords:** neurexin, neuroligin, synapse, neurodegenerative disorders, neuropsychiatric disorders, protein interactions

## Abstract

Synaptopathies are brain disorders characterized by dysfunctional synapses, which are specialized junctions between neurons that are essential for the transmission of information. Synaptic dysfunction can occur due to mutations that alter the structure and function of synaptic components or abnormal expression levels of a synaptic protein. One class of synaptic proteins that are essential to their biology are cell adhesion proteins that connect the pre- and post-synaptic compartments. Neurexins are one type of synaptic cell adhesion molecule that have, recently, gained more pathological interest. Variants in both neurexins and their common binding partners, neuroligins, have been associated with several neuropsychiatric disorders. In this review, we summarize some of the key physiological functions of the neurexin protein family and the protein networks they are involved in. Furthermore, examination of published literature has implicated neurexins in both neuropsychiatric and neurodegenerative disorders. There is a clear link between neurexins and neuropsychiatric disorders, such as autism spectrum disorder and schizophrenia. However, multiple expression studies have also shown changes in neurexin expression in several neurodegenerative disorders, including Alzheimer's disease and Parkinson's disease. Therefore, this review highlights the potential importance of neurexins in brain disorders and the importance of doing more targeted studies on these genes and proteins.

## Graphical abstract

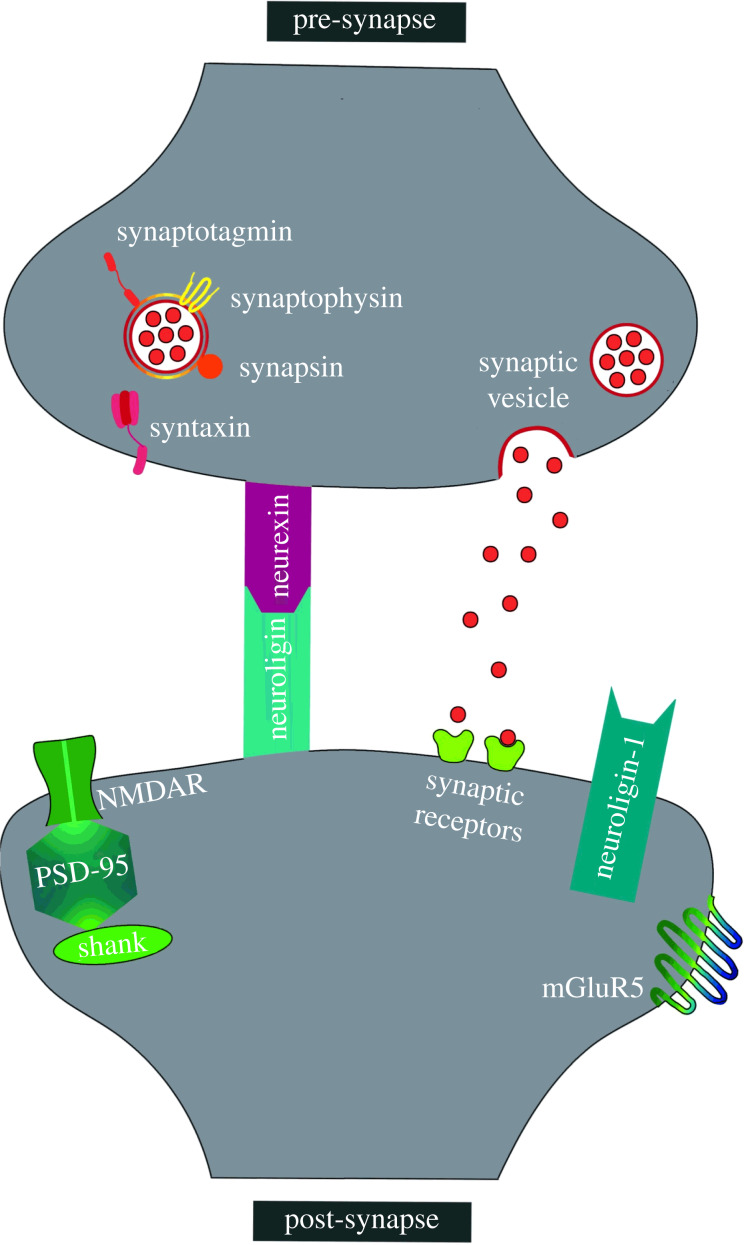

## 1. Introduction

There is accumulating evidence to suggest that synaptic dysfunction is present in both neuropsychiatric disorders, such as autism spectrum disorders (ASDs), schizophrenia and bipolar disorder (BD), and neurodegenerative disorders, such as Parkinson's disease (PD), Alzheimer's disease (AD) and Huntington's disease (HD) [1]. In fact, involvement of the synapse is such a prominent feature of the pathogenesis of various brain disorders that it has led to the coining of a specific term, ‘synaptopathies’. Indeed, in the case of PD, the involvement of synaptopathy as an initial and central event in the disease pathogenesis, which precedes neuronal damage, has been postulated [2]. Synaptic dysfunction can occur due to mutations that alter the structure and function of synaptic components or abnormal expression levels of a synaptic protein.

Synapses are specialized junctions between neurons that transmit information and they connect neurons into millions of ‘neural circuits’ that underlie all brain functions [3]. The information transmitted allows the nervous system to respond to external stimuli and controls bodily functions, behaviour, emotions and memories [4]. This system is tightly controlled and regulated, and even slight perturbations can lead to synaptic dysfunction. An important aspect of synapse biology is the cell adhesion molecules that connect pre- and post-synaptic compartments [5]. These interactions in the synaptic cleft help to maintain synapse structure by delineating mutual boundaries [6]. These proteins are also important in synapse plasticity as synaptic cell adhesion is able to regulate the remodelling of synapses [7]. Interestingly, they are also involved in trans-synaptic signalling [5]. Thus, these proteins are highly important in the organization of synaptic junctions and overall brain function.

**Figure 1.**
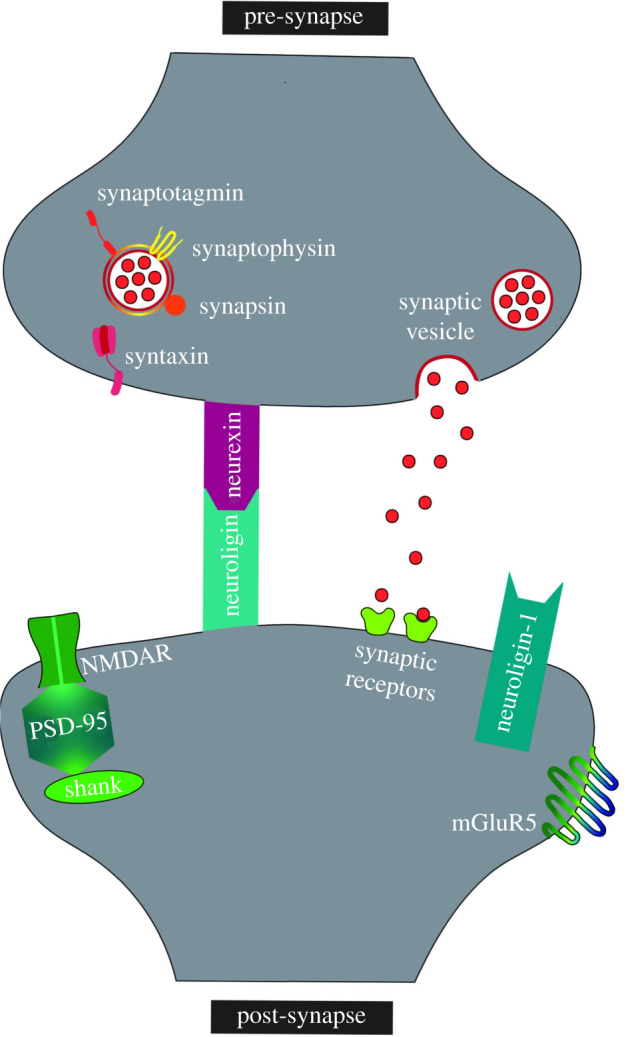
Location of neurexins and their binding partners, neuroligins, in the synapse. Several neurexin–neuroligin pathway proteins are shown as well as synaptic vesicle-binding proteins. NMDAR, *N*-methyl-d-aspartate receptor; mGluR5, metabolic glutamate receptor 5; PSD-95, post-synaptic density protein 95; Shank, SH3 and multiple ankyrin repeat domains protein.

Neurexins are one type of synaptic cell adhesion molecule. They are pre-synaptically localized and bind to neuroligins and other proteins in the post-synapse (figure 1). Neurexins and their common binding partners, neuroligins, have recently gained more pathological interest as variants in both have been associated with several neuropsychiatric disorders, including autism and schizophrenia [8]. This further suggests that synaptic dysfunction plays a role in the development of these disorders. Synaptic dysfunction is also known to occur in neurodegenerative disorders [9]; however, it was considered an endpoint of these disorders, due to the considerably later onset of clinical symptoms and progressive appearance of cognitive deficits. This dichotomy has, recently, been challenged by the creation of ‘disease-in-a-dish’ models for multiple central nervous system (CNS) pathologies [9]. This research has identified commonalities between developmental and degenerative disorders, at both the cellular and molecular level, with most of these common mechanisms meeting at the synapse level [9]. Indeed, our laboratory has, recently, found a novel variant (p.G849D) in the *NRXN2* gene which may be implicated in PD [10]. Therefore, we believe it is important to investigate the potential role of neurexins in various neuropsychiatric and neurodegenerative disorders.

In this review, we summarize some of the key physiological functions of the neurexin protein family and the protein networks they are involved in. We also examine the available published literature to determine what research has been done on neurexins in neuropsychiatric and neurodegenerative disorders. This analysis provides an overview on what progress has been made in understanding the roles of synaptic functioning in these disorders and reveals the gaps in knowledge in this field.

## 2. Structure and biological functions of neurexins

Neurexins were first identified using affinity chromatography when neurexin 1α was found in rat brain extract on a column of α-latrotoxin [11]. α-latrotoxin is a potent neurotoxin from black widow spider venom that stimulates synaptic vesicle exocytosis and induces massive neurotransmitter release [12]. This work has been continued by Südhof and co-workers [13] who have characterized the neurexin proteins and their binding partners, the neuroligins [14].

In mammals, the neurexins are encoded by three *NRXN* genes (*NRXN1-3*), each of which has both an upstream promoter that is used to generate the α-neurexins, and a downstream promoter that is used to generate the shorter β-neurexins [13,15]. Neurexins also undergo extensive alternate splicing at five splice sites, generating significant diversity of more than 2000 potential variants [13,16]. The fact that neurexin splice insert sequences and their positions are well conserved among neurexin genes and between species supports the idea that alternative splicing has important functional roles.

**Figure 2.**
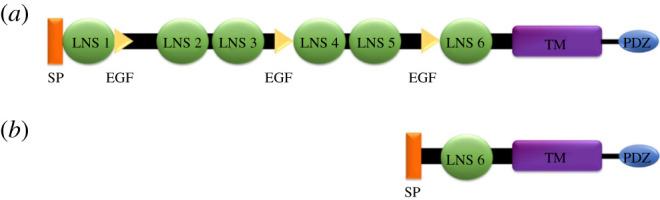
Structural domain organization of the α and β forms of neurexin. (*a*) α-neurexin. (*b*) β-neurexin. EGF, epidermal growth factor-like region; LNS, laminin/neurexin/sex hormone-binding domain; PDZ, PSD-95, DLG1, ZO-1 domain; SP, signal peptide; TM, transmembrane domain.

The neurexins are transmembrane proteins that consist of an extracellular region responsible for trans-synaptic interactions, a transmembrane domain and a smaller cytoplasmic domain named PSD-95, DLG1, ZO-1 binding domain (PDZ) that is involved in intracellular protein interactions and signalling (figure 2) [13]. α-neurexins are composed of six large extracellular laminin/neurexin/sex hormone-binding (LNS) globulin domains with three interspersed epidermal growth factor (EGF)-like regions (figure 2). β-neurexins are shorter and only have the sixth extracellular LNS domain and no EGF-like regions (figure 2). Only neurexin 1 protein structures (both α and β forms) have been solved experimentally in *Mus musculus, Rattus norvegicus* and *Bos taurus*. However, these structures have not yet been solved in humans.

Neurexins are localized pre-synaptically and are distributed to both excitatory and inhibitory synapses [8]. Their functions are mediated by their binding to neuroligins (figure 1). Neuroligins have five known isoforms and are expressed post-synaptically [17]. Consequently, neurexins and neuroligins form synaptic complexes in the synaptic cleft and have been found to control synapse formation, maturation, validation and function [17]. Various combinations of the different neurexins and neuroligin binding partners at synapses may also help determine synapse specificity through differential interactions between multiple splice variants and isoforms of these proteins [8].

**Figure 3.**
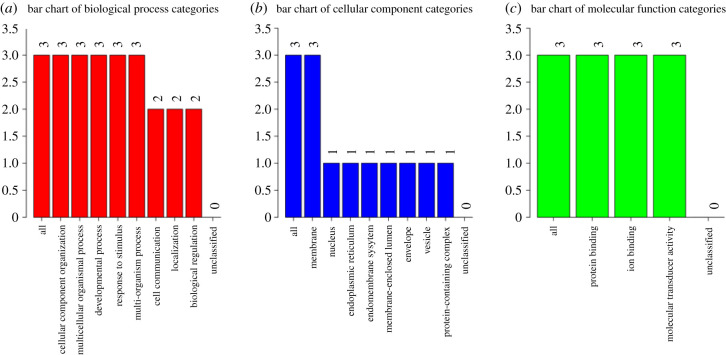
A summary of GO terms associated with neurexins 1–3. (*a*) Biological processes. (*b*) Cellular components. (*c*) Molecular functions. All: total number of proteins analysed. The number above each bar indicates the number of proteins assigned to that category. Figure generated by WebGestalt (http://www.webgestalt.org) [18].

Primarily, neurexins function to maintain synaptic organization. Gene ontology (GO) analysis by WebGestalt (http://www.webgestalt.org) [18] of the three neurexins indicates that they all function in protein binding, ion binding and possess molecular transducer activity (figure 3). They are also involved in cellular component organization, developmental processes, response to stimuli, cell communication and biological regulation. These processes thus demonstrate how neurexins are able to maintain synaptic organization but also show their multi-functional nature. As such, it is conceivable that disruptions in neurexins could be detrimental to their various functions and affect overall neuronal function and integrity.

## 3. Biological pathways and interacting partners of neurexins

To understand the broader biological pathways that the neurexins are involved in, protein–protein interaction network analysis and co-expression analysis was performed using the tools, STRING (https://string-db.org) [19] and GeneMania (https://genemania.org) [20]. STRING finds related genes by accessing the STRING database which contains experimental data and computational predictions. Data in STRING are weighted and integrated and a confidence score of 0–1 is calculated for all interacting protein partners. GeneMANIA finds proteins related to neurexins by leveraging functional association data, such as interactions, pathways, co-expression, co-localization and protein domain similarity. All functional data for the proteins observed in these networks were obtained from UniProt (https://www.uniprot.org) [21], unless otherwise stated, while pathway data were obtained from KEGG (https://www.kegg.jp) [22].

### 3.1. String analysis

**Figure 4.**
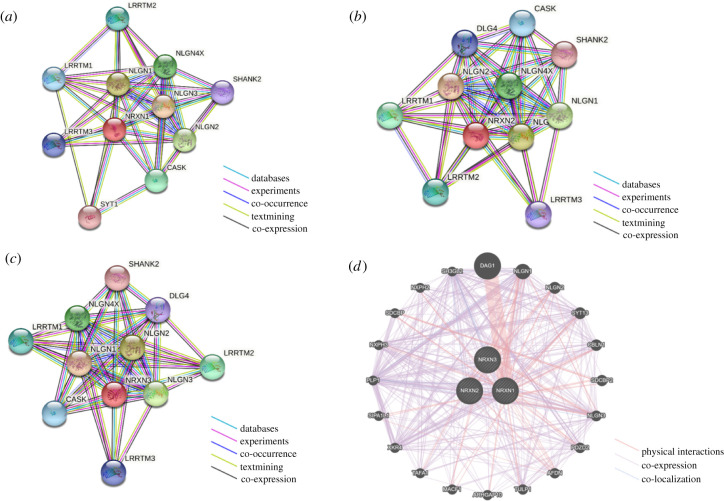
Protein interacting partners of neurexins 1–3. (*a*–*c*) STRING network of neurexins 1–3. Nodes represent gene-encoded proteins. Edges represent protein–protein associations. Connections between nodes represent the relationship between proteins. A bold line implies a higher confidence level. (*d*) GeneMANIA network of all three neurexins. Nodes represent gene-encoded proteins. Larger nodes indicate higher protein scores. Edges represent protein–protein associations. Connections between nodes represent the relationship between proteins. A bold line implies a higher confidence level.

Weighted string analysis was conducted on neurexin 1, 2 and 3 individually to determine their binding partners (figure 4*a*–*c*). Based on this analysis, there is strong evidence that neurexin 1 interacts with 10 proteins including calcium/calmodulin-dependent serine protein kinase (CASK), leucine-rich repeat transmembrane neuronal protein 1 (LRRTM1), LRRTM2, LRRTM3, neuroligin 1, neuroligin 2, neuroligin 3, neuroligin 4X, SH3 and multiple ankyrin repeat domains protein 2 (SHANK2) and synaptotagmin-1 with scores of 0.987, 0.983, 0.985, 0.975, 0.998, 0.997, 0.998, 0.997, 0.975, 0.974, respectively. Similarly, neurexins 2 and 3 also have 10 interactors each. There is strong evidence that neurexin 2 interacts with CASK, discs large homologue 4 (DLG4), LRRTM1, LRRTM2, LRRTM3, neuroligin 1, neuroligin 2, neuroligin 3, neuroligin 4X and SHANK2 with scores of 0.979, 0.977, 0.984, 0.983, 0.972, 0.998, 0.998, 0.998, 0.997 and 0.969, respectively. There is strong evidence that neurexin 3 interacts with CASK, DLG4, LRRTM1, LRRTM2, LRRTM3, neuroligin 1, neuroligin 2, neuroligin 3, neuroligin 4X and SHANK2 with scores of 0.978, 0.971, 0.979, 0.983, 0.971, 0.998, 0.997, 0.997, 0.997 and 0.969, respectively. The STRING analyses performed on the three neurexins identified interacting proteins with very high confidence scores since the lowest score across the analyses was 0.969. This means that there is strong experimental evidence that these proteins interact with one or more of the neurexins.

Analysis of the identified neurexin binding partners revealed many proteins important in the maintenance and functioning of synapses. Notably, variants in several of these proteins are implicated in neuropsychiatric and developmental disorders. Variants in neuroligin 1 and SHANK2 have been implicated in susceptibility to autism [23,24], while variants in neuroligin 3 have been implicated in Asperger syndrome and autism [25]. Variants in neuroligin 4X have been implicated in X-linked forms of Asperger syndrome, autism susceptibility and mental retardation [25–27]. Variants in CASK have been implicated in FG syndrome 4, an X-linked genetic disorder and mental retardation [28–32], variants in DLG4 have been implicated in intellectual developmental disorder 62 [33,34] and variants in synaptotagmin-1 have been implicated in Baker–Gordon syndrome [35].

Furthermore, binding partners of these proteins as well as the pathways they occur in could also give insight into the development of disease. CASK binds to amyloid precursor protein and neuroligin 1 binds to amyloid-β, both of which are important in AD. LRRTM3 is also a known positive regulator of amyloid-β formation. Notably, *LRRTM3* may be considered a candidate gene for late-onset AD as it promotes the processing of amyloid precursor protein which leads to toxic amyloid-β accumulation [36]. DLG4 is involved in dopamine receptor binding and synaptotagmin-1 regulates dopamine secretion. The loss of dopamine functioning is crucial in PD. Indeed, DLG4 is involved in several pathways of neurodegeneration (in multiple diseases), the HD pathway as well as cocaine addiction.

### 3.2. GeneMANIA

GeneMANIA analysis was performed on the neurexins to reveal further potential protein–protein interactions (figure 4*d*; electronic supplementary material, table S1). We performed the analysis by selecting only proteins with stronger evidence of neurexin interactions, such as interactions with physical evidence, and evidence from co-expression and co-localization studies.

All of the binding partners observed by STRING analysis were still present; however, more interacting proteins were also identified. These proteins have more diverse functions but still function in overall synapse maintenance.

This analysis further identified afadin (AFDN), Rho GTPase activating protein 10 (ARHGAP10), cerebellin 1 (CBLN1), dystroglycan (DAG1), microtubule actin cross-linking factor 1 (MACF1), neurexophilin-2, neurexophilin-3, PDZ domain-containing protein 2 (PDZD2), proteolipid protein 1 (PLP1), syndecan binding protein 1 (SDCBP), SDCBP2, SH3 domain-containing GRB2-like protein 2 (SH3GL2), signal-induced proliferation-associated 1-like protein 1 (SIPA1L1), synaptotagmin-13 (SYT13) TAFA chemokine-like family member 1 (TAFA1), TUBB-like protein 1 (TULP1) and XK-related protein 4 (XKR4) as interactors of one or more neurexin proteins. AFDN, ARHGAP10, MACF1 and SIPA1L1 are all involved in actin filament binding/organization, while PDZD2, SDCBP and SDCBP2 are involved in cell binding and cytoskeletal organization. Dysregulation of any of these proteins could thus affect cell adhesion and binding at the synapse. In addition, variants in MACF1 have been implicated in lissencephaly 9 with complex brainstem malformation [37]. CBLN1 is essential for synapse integrity and plasticity and its disruption could lead to synapse dysfunction. DAG1 has multiple functions, such as laminin and basement membrane assembly, cell survival, peripheral nerve myelination, nodal structure and cell migration. Variants in DAG1 have been implicated in both type A and type C muscular dystrophy–dystroglycanopathy [38–41]. Muscular dystrophies are genetic disorders characterized by the degeneration of skeletal muscle. Type C muscular dystrophy–dystroglycanopathy affects the limb-girdle area [40], while type C is congenital with brain and eye anomalies [39]. Neurexophilin-2 and neurexophilin-3 are both ligands for α-neurexins and are involved in the neuropeptide signalling pathway. Disruption of these proteins could, therefore, affect neurotransmitter release and the subsequent signalling. PLP1 is the major myelin protein in the CNS and is important for maintaining the structure of myelin. Disruption of this protein could, therefore, negatively affect the downstream myelination of neurons, as is seen in multiple sclerosis (MS). Interestingly, PLP1 is also involved in the development of the substantia nigra, the main brain region affected by PD. Therefore, PLP1 alterations could also lead to disruptions in this brain region. SH3GL2 has been implicated in synaptic vesicle endocytosis, while synaptotagmin-13 may be involved in transport vesicle docking to the plasma membrane. Dysregulation of these proteins could thus affect neurotransmitter functioning. TAFA1 is involved in the modulation of neural stem cell proliferation and differentiation; therefore, dysregulation of this protein could result in developmental disorders. TULP1 is required for normal development of photoreceptor synapses. Variants in TULP1 are associated with Leber congenital amaurosis [42,43] and retinitis pigmentosa [42,44–47]. However, this protein is also involved in actin filament binding, therefore, its dysregulation could also affect cell adhesion and binding at the synapse. Not much is known about XKR4 except that it is involved in apoptosis during development. Therefore, its dysregulation could also possibly result in developmental disorders.

GO terms and physiological/pathway information on all binding partners identified by STRING and GeneMANIA are available in electronic supplementary material, figure S1 and table S1.

## 4. Role of neurexins in neuropsychiatric disorders

Literature-based searches using neurexin as a search term identified several studies that reported an association of neurexins in various neuropsychiatric disorders. The main findings of these studies are reported in table 1 and are summarized below.

**Table 1.** List of studies that have implicated neurexin genes in neuropsychiatric disorders. AAV, adeno-associated virus; AGRE, Autism Genetic Resource Exchange; Array-CGH, array comparative genomic hybridization; CBDB, Clinical Brain Disorders Branch; CIBERSAM, Centro de Investigación Biomédica en Red de Salud Mental; CNV, copy number variation; EMAS, epilepsy with myoclonic-atonic seizures; GWAS, genome-wide association study; hESC, human embryonic stem cell; iN, induced neuron; iPSC, induced pluripotent stem cell; KO, knockout; LC-MS/MS, liquid chromatography mass spectrometry/mass spectrometry; mESC, mouse embryonic stem cell; mTLE, mesial temporal lobe epilepsy; NGS, next-generation sequencing; NIMH, National Institute of Mental Health; RT–PCR, reverse transcriptase–polymerase chain reaction; SNP, single nucleotide polymorphism; SSC, Simons Simplex Collection; STEP-BD, Systematic Treatment Enhancement Program for Bipolar Disorder; WT, wild-type.

disorder/disease process	neurexin gene	type of study	methods	main finding	reference
autism spectrum disorder (ASD)	NRXN1	genetic analysis	used SSC samples and the SSC database to extract ‘trios’ consisting of a mother, father and an ASD-affected child	a de novo CNV in *NRXN1* was discovered in a large cohort of families with a single ASD-affected child and at least one unaffected sibling	[48]
			performed genetic analyses to identify CNVs		
ASD	NRXN1	GWAS	1174 families from SSC were genotyped	rare de novo events/CNVs at *NRXN1* are strongly associated with autism	[49]
			identified CNVs and de novo events		
ASD	NRXN1β	genetic analysis	86 patients with both ASD and mental retardation	four novel mutations in *NRXN1β* were identified by sequencing the exon of the gene in cases with autism and mental retardation	[50]
			The coding sequence of the *NRXN1β* gene was analysed by PCR		
ASD	NRXN1	genetic analysis	313 ASD patients and 500 healthy controls from a Chinese autism cohort were recruited	22 variants in the *NRXN1* gene were discovered in the Chinese Han population; one SNP (rs2303298) was significantly associated with a risk of autism in this cohort	[51]
			performed genomic DNA sequencing		
ASD	NRXN1	genetic analysis	2478 ASD individuals from SSC and 719 ASD individuals from AGRE	recurrent CNVs in *NRXN1* are enriched in autism.	[52]
			580 controls from ClinSeq and NIMH		
			used a custom microarray to analyse CNVs		
ASD	NRXN1, 2 and 3	cell culture	iPSCs were produced from probands and unaffected family members	neurexin 1, 2 and 3 mRNA is overexpressed in patient-derived iPSCs and differentiated organoids	[53]
			iPSCs underwent neuronal differentiation to organoids		
			RNA sequencing was performed on both iPSCs and differentiated organoids		
ASD	NRXN1	genetic analysis	2591 families from SSC were genotyped	*NRXN1* is an ASD risk gene	[54]
			identified CNVs, de novo deletions and ASD risk genes		
ASD	NRXN1 and 2	animal study	RNA was isolated from the whole brain of age-matched monoamine oxidase A KO mice and wild-type mice	neurexin 1 and 2 are downregulated in monoamine oxidase A KO mice	[55]
			Microarrays were used to determine gene expression levels		
ASD	α-NRXNs	animal and cell culture study	transfected *C. elegans* strains and HEK-293 cells with plasmids expressing NRXN1α and different α2δ subunits	changes in α-neurexin binding to α2δ-3 subunits of N-type calcium channels could be important in some forms of autism spectrum disorders	[56]
			performed co-immunoprecipitation and pull-down assays		
ASD	NRXN2	genetic analysis	142 ASD patients and 94 non-syndromic controls	observed a frameshift mutation in *NRXN2* exon 12 in a patient with ASD inherited from a father with severe language delay	[57]
			sequenced NRXN 1,2 and 3 genes		
ASD	NRXN2	genetic analysis	recruited a patient with speech problems, autistic traits and pancreatic gastrinoma	a de novo 0.57 Mb microdeletion was observed in chromosome 11q13.1, including *NRXN2*	[58]
			performed array-CGH		
ASD	NRXN2	animal study	used previously collected human faecal samples from typically developing children and children with ASD	mice colonized by microbiota from ASD patients showed differential splicing of *NRXN2*	[59]
			C57BL/6 J weanlings were colonized with human faecal samples		
			brain tissue RNA was extracted and sequenced		
bipolar disorder (BD)	NRXN3	GWAS	obtained participants from a family study of mood disorders in Taiwan (2008–2012)	*NRXN3* shows a significant association with bipolar disorder	[60]
			performed a multi-stage GWAS		
BD	NRXN1	genetic analysis	obtained patient genotyping and clinical data from STEP-BD	*NRXN1* may affect the long-term treatment outcome of bipolar disorder	[61]
			analysed data to determine the effect of individual markers on phenotypes		
borderline personality disorder (BPD)	NRXN3	association study	1439 heroin-dependent BPD cases and 507 neighbourhood controls	several *NRXN3* SNPS were nominally associated with BPD phenotype in heroin-dependent cases	[62]
			genotyped NRXN3 SNPs and performed an association analysis		
epilepsy	NRXN1	microarray analysis	obtained 53 biopsy specimens from mTLE patients	neurexin 1 is differentially expressed in non-responder and responder mTLE patients to the antiepileptic drug Levetiracetam	[63]
			performed microarray analysis	lower levels of neurexin 1 are observed in responder patients	
epilepsy	NRXN1	genetic testing	77 patients were identified at Children's Hospital Colorado	a 2p16.3 deletion, which includes the first five exons of the *NRXN1* gene, was identified in an 8-year-old male EMAS patient	[64]
			various genetic tests were conducted		
epilepsy/seizures	NRXN2α	animal study	treated adult Wistar rats with kainite or pentylenetetrazole to induce seizures	following kainate- and pentylenetetrazole-induced seizures in rats, neurexin 2α expression increased in the dentate gyrus of the hippocampus	[65]
			isolated total RNA from whole-rat brains and hippocampi		
			performed RT–PCR to determine the levels of different NRXNs		
fragile X syndrome	NRXN3	animal study	used male and female WT and FMR1 KO mice (4–6 per experiment)	there is increased neurexin 3 mRNA in female fragile X mice, but decreased neurexin 3 mRNA in male fragile X mice	[66]
			analysed brain sections using riboprobes for NRXN1, 2 and 3 and NLGN 1, 2 and 3		
major depressive disorder (MDD)	NRXN1, 2 and 3	animal study	81 healthy Sprague–Dawley rats were subjected to various mild stress factors	neurexin 1, 2 and 3 were not differentially expressed in a rat chronic mild stress model of depression	[67]
			extracted proteins from hippocampal post-synaptic density fractions		
			analysis by LC-MS/MS		
neuropsychiatric disorders	NRXN1	cell culture	cultured cortical neurons from NRXN1*α* KO mice	heterozygous NRXN1 mutations are able to selectively impair neurotransmitter release and increase the levels of the synaptic scaffolding protein, CASK in human iNs but not in the cortical neurons of NRXN1*α* KO mice	[68]
			introduced conditional NRXN1 mutations into hESCs using AAV recombination, and differentiated them into human iNs		
			analysed neuronal development, synapse formation and neurotransmitter release		
schizophrenia (SCZ)	NRXN1	microarray analysis	obtained brain tissue from 12 SCZ patients and 10 controls	the expression of neurexin 1 was not significantly different between the schizophrenic and control subjects	[69]
			extracted total RNA		
			performed a microarray analysis		
SCZ	NRXN1	genetic analysis	selected 45 male and 48 female proband-parent trios from a sample of 600 Bulgarian SCZ trios	observed a 0.25 Mb deletion on 2p16.3 in both the proband and affected sibling which disrupts *NRXN1*	[70]
			performed Array-CGH		
SCZ	NRXN1	GWAS	3063 SCZ patients and 2847 controls from CIBERSAM	the rs3850333 SNP in the *NRXN1* gene was close to the significant threshold in a GWAS of schizophrenia in Spain	[71]
			performed a GWAS at 95 SNPs		
SCZ	NRXN1	genetic analysis	obtained DNA of 635 SCZ patients and 635 controls from the CBDB Sibling Study	*NRXN1* deletions are more frequent in schizophrenia patients	[72]
			genotyped samples and analysed them for CNVs and deletions	there is incomplete penetrance of *NRXN1* deletions in families with schizophrenia	
SCZ	NRXN1	genetic analysis	data from 572 SCZ patients and 551 controls were used to select genes for sequencing	missense variants at *NRXN1* may be protective against schizophrenia susceptibility	[73]
			153 SCZ patients and 153 controls were sequenced for 21 chosen genes using NGS		
SCZ	NRXN1	cell culture	isolated primary rat neurons from hippocampi	overexpressing Caveolin-1, a potential therapeutic for schizophrenia, in neurons increased expression of proteins involved in synaptic plasticity (PSD95, synaptobrevin, synaptophysin, neurexin 1 and syntaxin 1) as well as DISC1	[74]
			differentiated human neurons derived from human iPSCs		
			overexpressed Caveolin-1 in both cell types		
			western blotting was used to measure the expression of proteins involved in synaptic plasticity as well as DISC1, an SCZ-associated protein		
SCZ	NRXN1	animal study	generated iPSCs from 5 childhood-onset SCZ patients and 4 controls	neurexin 1 was downregulated in chimeric mice produced from iPSCs derived from patients with childhood-onset schizophrenia	[75]
			differentiated iPSCs into glial cells		
			transplanted glial cells into mice via injection into the corpus collosum		
			performed molecular analyses on both the differentiated glial cells and chimeric mice		
SCZ	NRXN1	GWAS	obtained genetic data and treatment response data of 302 SCZ patients treated with lurasidone and 117 SCZ patients treated with a placebo from two clinical SCZ trials	*NRXN1* is associated with antipsychotic response to lurasidone in schizophrenia patients	[76]
			performed a GWAS		
SCZ	NRXN1	cell culture	generated iPSCs from 3 NRXN1 deletion SCZ patients and 3 controls and differentiated them into human iNs	heterozygous NRXN1 deletions impair neurotransmitter release and synaptic function, and increase the levels of the synaptic scaffolding protein, CASK in human iNs but not mESCs generated from NRXN1 KO mice	[77]
			generated mESCs from NRXN1 KO mice		
			analysed neuronal development, synapse formation and neurotransmitter release		
SCZ and other neuropsychiatric disorders	NRXN1	genetic analysis	recruited a family with multiple neuropsychiatric disorders	two rare deletions upstream of the *NRXN1* gene (2p16.3) segregate with schizophrenia, schizophreniform disorder, and affective disorder in a family	[78]
			the proband has SCZ, while other family members have mental retardation, schizophreniform disorder and affective disorder		
			genotyped the proband and eight family members		

### 4.1. Human studies

*NRXN1* has been well documented for its association with ASDs [54]. Several genetic analyses of families and populations of people with ASD have shown that copy number variations (CNVs) and de novo mutational events at the *NRXN1* locus are enriched in ASD [48,49,51,52,54]. In one study, *NRXN1* was sequenced in cases of ASD with mental retardation [50]. Mutations (c.–3G > T in the Kozak region, c.3G > T at the initiation codon (p.M1), p.R375Q and p.G378S) were found in the *NRXN1β* coding region thereby potentially implicate synapse dysfunction an important determinant in ASD [50].

The first evidence for a potential role of *NRXN2* in ASD was provided by a report of a frameshift mutation within *NRXN2* exon 12 (c.2733delT) in a boy with ASD and his father who had severe language delay [57]. This mutation results in a truncated neurexin 2α protein that lacks the binding sites for the established post-synaptic binding partners LRRTM2 and neuroligin 2 [57]. Subsequently, a 21-year-old man with a clinical phenotype including autistic traits, such as speech and language deficits and pathological insistence on routine, was reported to have a 570 kb de novo deletion of 24 genes at chromosome 11q13.1, including *NRXN2* [58].

Using microarray analyses on RNA extracted from brain tissue, Mirnics *et al.* [69] did not observe a difference in neurexin 1 expression between schizophrenia (SCZ) and control samples. However, since then, a link between neurexin 1 and SCZ has been reported in other studies. One study reported that *NRXN1* deletions are more common in those with SCZ; however, it also found that there was incomplete penetrance of these deletions in families with SCZ [72]. Kirov *et al.* [70] observed a deletion in an SCZ patient at 2p16.3 that disrupts *NRXN1* and predicted that it was highly likely to be pathogenic. Also, *NRXN1* deletions were shown to segregate with several neuropsychiatric disorders in a study of a complex family [78]. The proband had SCZ and other members of his family had mental retardation, schizophreniform disorder and affective disorder [78]. After genotyping the proband and eight family members, they found two rare deletions upstream of the *NRXN1* gene (2p16.3) that co-segregate with these disorders [78]. Notably, this shows that deletions in *NRXN1* may manifest as multiple neuropsychiatric phenotypes.

Angione *et al.* [64] implicated *NRXN1* in epilepsy. They identified a 2p16.3 deletion in an 8-year-old male patient diagnosed with epilepsy showing symptoms of myoclonic-atonic seizures (EMAS) [64]. This deletion included the first five exons of the *NRXN1* gene [64].

*NRXN* genes may also be involved in treatment response. In one study, it was found that variants in *NRXN1* may affect the long-term treatment outcome of patients with BD by modulating the effects of antipsychotics [61]. In a study of Levetiracetam resistance, an antiepileptic drug, Grimminger *et al.* [63] found that neurexin 1 is differentially expressed in non-responder and responder patients with mesial temporal lobe epilepsy (mTLE), whereby lower levels of neurexin 1 were observed in responder patients.

#### 4.1.1. Association studies

A genome-wide association study (GWAS) by Liu *et al.* [51] specifically examined *NRXN1* in an autism cohort of the Chinese Han population and discovered 22 variants that were associated with ASD. In this cohort, one SNP (rs2303298) was also significantly associated with a risk of developing ASD [51]. Furthermore, a GWAS of SCZ in Spain showed that a *NRXN1* single nucleotide polymorphism (SNP) (rs3850333) was close to the significance threshold [71], while another GWAS in American patients of European or African ancestry showed that *NRXN1* is associated with antipsychotic response to lurasidone in SCZ patients [76]. Additionally, an association study on Spanish SCZ patients showed that missense mutations in *NRXN1* may actually protect against susceptibility to SCZ [73].

In a Taiwanese GWAS study, a significant association between *NRXN3* and BD was found [60]. And finally, an association study on Australian borderline personality disorder (BPD) patients showed that several *NRXN3* SNPS were nominally associated with BPD in heroin-dependent cases [62].

### 4.2. *In vitro* and *in vivo* models of disease

Functional *in vitro* and *in vivo* studies have also found evidence for the roles of neurexins in ASD. Monoamine oxidase A knockout (KO) mice, which are an animal model for autism, exhibited downregulated levels of both neurexin 1 and neurexin 2 [55]. Furthermore, mice colonized with the microbiota of ASD patients showed differential splicing of *NRXN2* [59]. Another animal study showed that changes in the binding of α-neurexins to N-type calcium channels could be important for some forms of ASD as it mediates synaptic inhibition [56]. Finally, a study using ASD patient-derived induced pluripotent stem cells (iPSCs) and differentiated organoids showed that neurexin 1, 2 and 3 mRNA is overexpressed in these samples [53].

One study examined neurexins in Fragile X syndrome, a genetic disorder with features similar to ASD, and characterized by the silencing of the *FMR1* gene [79]. Individuals with Fragile X experience a range of neurodevelopmental problems, such as learning disabilities and cognitive impairment, and males are usually more severely affected. Using *FMR1* KO mice, researchers probed brain sections to determine the levels of neurexin 1, 2 and 3 [66]. Interestingly, they found that neurexin 3 mRNA levels are increased in female mice but decreased in male mice and predicted that this may help explain the sex difference observed in this disorder [66].

In an animal study of SCZ, neurexin 1 was found to be downregulated [75]. This study generated iPSCs from patients with childhood-onset SCZ, differentiated them into glial cells and injected the glial cells into mice to form chimeric mice as a model organism [75]. Interestingly, an *in vitro* study of SCZ showed that overexpressing Calveolin-1, a potential therapeutic for SCZ, actually increased the levels of neurexin 1 as well as other proteins involved in synaptic plasticity [74].

Neurexin 2α has been implicated in epilepsy and, more specifically, in seizures. Researchers observed an increase in neurexin 2α expression in the dentate gyrus of the hippocampus in an induced-seizure mouse model [65]. Finally, in one study, a rat chronic mild stress model of depression was used to determine if neurexin expression was altered in major depressive disorder; however, no change in neurexin 1, 2 or 3 levels was observed [67].

So far, there have been two studies validating the effect of NRXNs *in vitro*, both by Pak *et al.* [68,77]. These studies cultured human stem cells as well as mice cells generated from *NRXN1* KO mice. The first study introduced two conditional NRXN1 mutations previously seen in a range of neuropsychiatric disorders, including ASD and SCZ, into human embryonic stem cells (hESCs) using adeno-associated virus recombination and differentiated them into human-induced neurons (iNs) [68]. These cells were compared to cortical neurons generated from NRXN1α KO mice [68]. The second study generated iPSCs from three NRXN1 deletion SCZ patients and three controls, and again differentiated them into human iNs [77]. These cells were compared to mouse embryonic stem cells (mESCs) from NRXN1 KO mice [77]. Both studies showed that heterozygous NRXN1 deletions were able to impair neurotransmitter release and synaptic function, and increase the levels of the synaptic scaffolding protein, CASK, in human iNs but not in mice cells [68,77]. Therefore, these studies provide evidence that NRXN1 deletions exhibit a major synaptic transmission phenotype in humans and are thus meaningful at a pathophysiological level.

In summary, these studies demonstrate a link between *NRXN*s and neuropsychiatric disorders such as ASD and SCZ, especially involving full or partial deletions of these genes. *NRXN*s have also been associated with BD and BPD. In addition, protein expression studies have shown changes in neurexin expression in animal models of epilepsy/seizures and Fragile X syndrome.

## 5. Role of neurexins in neurodegenerative disorders

Additionally, literature-based searches provided proof for the involvement of neurexins in various neurodegenerative disorders, and these studies are listed in table 2 and discussed below.

**Table 2.** List of studies that have implicated neurexin genes in neurodegenerative disorders and ageing. 6-OHDA, 6-hydroxydopamine; ACP-RT–PCR, annealing control primer reverse transcriptase–polymerase chain reaction; ADNI, Alzheimer's disease neuroimaging initiative; AMPA4, GluA4-containing glutamate; CSF, cerebrospinal fluid; EAE, experimental autoimmune encephalomyelitis; ELISA, enzyme-linked immunosorbent assay; FTD-GWAS, frontotemporal dementia genome-wide association study; GEO, gene expression omnibus; GWAS, genome-wide association study; HIV, human immunodeficiency virus; HYPERGENES, European Network for Genetic-Epidemiological Studies; LC-MS/MS, liquid chromatography mass spectrometry/mass spectrometry; LC-SRM, liquid chromatography single reaction monitoring; MAP, Rush Memory and Ageing Project; MR, magnetic resonance; MRI, magnetic resonance imaging; NPTX2, neuronal pentraxin 2; ONIND, other non-inflammatory neurological disease; PCDH8, protocadherin-8; PPMI, Parkinson's Progression Markers Initiative; qRT-PCR, quantitative real-time PCR; RAP-PCR, reverse arbitrarily primed PCR; rMOG, rat myelin oligodendrocyte glycoprotein; RRMS, relapsing–remitting MS; RT–PCR, reverse transcriptase–PCR; SNP, single nucleotide polymorphism; UV-CLIP, ultraviolet cross-linking and immunoprecipitation; WES, whole-exome sequencing.

disorder/disease process	neurexin gene	type of study	methods	main finding	reference
Alzheimer's disease (AD)	NRXN3	GWAS of brain structure	obtained neuroimaging and genetic data from 818 subjects as part of ADNI	*NRXN3* (*KIAA0743*) is associated with temporal lobe structure in AD patients	[80]
			performed a GWAS with 546,314 SNPs using temporal lobe and hippocampal volume as quantitative phenotypes		
AD	NRXN1	protein expression analysis	collected CSF samples from 10 AD patients and 15 healthy controls	the concentrations of the synaptic proteins neurexin 1 and neuronal PTX1, as well as neurofascin, were significantly lowered in AD CSF	[81]
			analysis using LC-MS/MS		
AD	NRXN2α	protein expression analysis	collected blood and CSF samples from 28 AD patients and 28 controls	significantly lower levels of the synaptic proteins NPTX2, AMPA4, neuroligin 1 and neurexin 2α in the plasma neuron-derived exomes correlate with AD progression	[82]
			extracted plasma neuron-derived exomes		
			CD81, NPTX2, AMPA4, neuroligin 1 and neurexin 2α proteins were quantified using ELISAs		
AD	NRXN1, 2 and 3	protein expression analysis	collected CSF samples from six AD patients and five non-AD patients	Aβ_42_ fibrils in AD CSF are involved in binding to proteoglycans, growth factors and neuron-associated proteins, such as neurexin 1, 2 and 3	[83]
			binding assays were performed to determine which proteins in CSF bind to Aβ_42_ fibrils and/or protofibrils		
AD	NRXN3	transcriptome and RNA expression analysis	selected data from 263 AD patients and 151 non-demented controls sampled from the religious orders study	neurexin 3 expression is downregulated in AD	[84]
			performed RNA expression profiling		
AD	NRXN2α and NRXN3α	protein expression analysis	collected CSF samples from AD patients and cognitively normal controls (three stage study with different *n* for each stage)	levels of neurexin 2α and neurexin 3α, as well as other synaptic proteins are decreased in preclinical AD CSF	[85]
			performed LC-MS/MS and LC-SRM		
AD and ageing	NRXN1, 2 and 3	microarray analysis	obtained frozen brain samples from 26 AD cases and 55 non-AD controls from National Institute on Ageing Alzheimer's disease brain banks	SYNAPTIC proteins, including neurexin 1, 2 and 3, undergo altered expression in ageing and AD	[86]
			used microarrays to evaluate expression profiles of 340 synaptic genes		
AD and ageing	NRXN3	animal study	mice were divided into four groups, with four mice in each group: memory intact AD-transgenic mice, memory impaired AD-transgenic mice, memory intact aged mice and memory impaired aged mice	neurexin 3 is downregulated in AD-transgenic mice with impaired memory, but not in normal aged mice with impaired memory	[87]
			performed proteomics on the hippocampus of each mouse		
AD and ageing	NRXN1 and 3	microarray analysis	performed a microarray analysis on 47 post-mortem brain samples from cognitively intact aged individuals from the MAP study	neurexin 1 and 3 have decreased expression in ageing and AD but have increased expression in association with late-life physical activity	[88]
			identified 48 microarrays from the public GEO: 16 young cases, 18 cognitively intact aged cases and 14 AD cases		
			analysed data to identify genes related to physical activity, ageing and AD		
ageing	NRXN3	animal study	cerebella were removed from three adult C57BL/6 J mice and three aged C57BL/6Jnia mice	neurexin 3 is downregulated in the cerebellum of aged mice	[89]
			RNA was extracted and sequenced		
ageing	NRXN2	methylation analysis	monocytes were purified from PBMCs	CpG sites associated with *NRP1*, *NRXN2* and miR-29b-2 are hypomethylated in monocytes during ageing	[90]
			analysis of methylation was performed on genomic DNA from monocytes		
ageing	NRXN1	animal study	28 Swiss albino mice were divided into four groups by age: young, adult, middle age and old	neurexin 1 and neuroligin 3 are differentially expressed in cerebral cortex and hippocampus during different stages of ageing, which might be responsible for alterations in synaptic plasticity during ageing	[91]
			molecular techniques were used to analyse neurexin 1 and neuroligin 3 expression		
ageing	NRXN2 and 3	transcriptome analysis	collected data of 2202 post-mortem human brain samples of neurologically healthy individuals with different ages	neurexin 2 and 3 are downregulated in ageing	[92]
			Calculated signal expression of genes		
amyotrophic lateral sclerosis (ALS)	NRXN1	cell culture and expression analysis	performed UV-CLIP experiments on SH-SY5Y cells to find TDP-43 targets	neurexin 1 and other TDP-43 targets are dysregulated in ALS	[93]
			validated these results on lumbar spinal cords from 4 ALS patients and 4 controls using RT-PCR		
HIV encephalitis	NRXN1	microarray analysis	received cortical brain tissue from 13 HIV patients: eight with HIV encephalitis and five without	neurexin 1 is downregulated in HIV encephalitis	[94]
			extracted total RNA		
			performed microarray analysis		
mild cognitive impairment (MCI)	NRXN1 and 2	microarray analysis	obtained frozen brain samples from 16 MCI cases, 25 AD cases and 24 aged controls from National Institute on Aging Alzheimer's Disease brain banks	neurexin 1 and 2 are upregulated in MCI	[95]
			extracted total RNA		
			performed a microarray analysis		
MCI and AD	NRXN1	association study	obtained brain MR images of 400 MCI subjects, 400 AD subjects and 200 aged controls from the ADNI database	neurexin 1 expression is associated with longitudinal phenotypes in MCI, but not in AD	[96]
			obtained genotype data for 510 of these subjects from the ADNI database		
			performed an association study		
multiple sclerosis (MS)	NRXN3	animal study	induced EAE in 17 rats by injecting rMOG	neurexin 3 is downregulated in grey matter of EAE-induced rats	[97]
			six control rats were treated with saline		
			extracted total RNA		
			used a cDNA expression array		
MS	NRXN2α	protein expression analysis	collected CSF samples from 37 RRMS patients, 50 patients with ONIND and patients with non-neurological (orthopaedic) diseases	neurexin 2α in CSF is a potential biomarker for MS	[98]
			analysis using LC-MS/MS		
MS	NRXN1	genetic analysis	collected blood from a female patient with RRMS	overexpression of neurexin 1 by mutant MIR8485 leads to calcium overload in pre-synapses. This could induce neurodegeneration in MS	[99]
			performed WES and screened for mutations		
MS	NRXN1	cell culture	treated THP-1 cells with ceramides to induce hypermethylation of DNA	ceramide-induced hypermethylation of DNA was associated with decreased transcript levels of neurexin 1 in cultured human monocytes	[100]
			isolated genomic DNA		
			measured levels of neurexin 1, FZD7 and TP63 using qRT-PCR		
neurodegeneration	NRXN3	animal study	45 DA(RT1^av1^) and 45 PVG(RT1^c^) adult rats	neurexin 3 is downregulated in rats with axonal damage caused by ventral root avulsion	[101]
			subjected rats to ventral root avulsion		
			extracted total RNA		
			used a cDNA expression assay and performed RT–PCR		
neurodegeneration	NRXN3	animal study	three experimental groups with five ICR mice each	neurexin 3 is downregulated in the hippocampus of mice treated with kainic acid, an inducer of neurodegeneration	[102]
			injected kainic acid into ICR mice		
			extracted total RNA from the hippocampus		
			performed ACP-RT-PCR and sequenced the PCR products		
neurodegeneration	NRXN1	bioinformatics and cell culture	analysed cross-linking, immunoprecipitation and sequencing data from the ArrayExpress archive to identify RNAs bound to TDP-43 in human and mouse brains	a novel TDP-43 binding miRNA, miR-NID1 (miR-8485), represses neurexin 1 expression and may play a role in neurodegeneration	[103]
			quantitative RT–PCR was used to measure mRNA expression		
neurodegeneration	NRXN1β	cell culture	transfected rat hippocampal neurons to overexpress acetylcholinesterase	excessive glycosylated acetylcholinesterase could competitively disrupt neurexin 1β-neuroligin junctions and impair the integrity of glutamatergic synapses	[104]
			performed a co-immunoprecipitation assay with neurexin 1 and acetylcholinesterase		
			co-transfected HEK-293 cells to express neurexin 1β and neuroligin 1 and cultured these cells in acetylcholinesterase conditioned media		
			performed a co-immunoprecipitation assay with neurexin 1β and neuroligin 1		
neurotoxicity	NRXN3β	animal study	groups of 3 Sprague–Dawley rats were treated with sarin via intra-muscular injection	sarin exposure causes a persistent downregulation of neurexin 1β and breakdown of the blood–brain barrier	[105]
			rats were sacrificed 15 min or 3 months after sarin exposure		
			dissected brains and extracted total RNA		
			performed a microarray analysis		
neurotoxicity	NRXN2α	animal study	wild-type zebrafish were repeatedly exposed to domoic acid via intracoelomic injection	neurexin 2α was upregulated in zebrafish two weeks after exposure to domoic acid, a neurotoxin	[106]
			dissected brains and extracted total RNA		
			performed a microarray analysis		
Parkinson's disease (PD)	NRXN1	cell culture	cultured SH-SY5Y cells and primary mouse mesencephalic cells	downregulation of neurexin 1 mRNA and protein was observed in the 6-OHDA-induced cell culture models of PD	[107]
			treated cells with 6-OHDA		
			performed RAP-PCR and analysed the PCR products using RT–PCR and qRT–PCR		
PD	NRXN2	animal study	transgenic mice were assigned to 4 treatment groups with 20 mice per group	transgenic mice overexpressing α-synuclein have increased levels of neurexin 2	[108]
			cholesterol oximes were administered in food pellets	chronic administration of cholesterol oximes to these mice decreased neurexin 2 levels	
			TH+ neurons were isolated from the substantia nigra and subjected to a transcriptome analysis		
PD	NRXN3	genetics analysis	obtained genomic data of 29 PD samples and 18 controls from the GEO database	genes related to nerve function, such as PCDH8 and neurexin 3, are downregulated in PD brain tissue samples	[109]
			analysed the data to identify disease-related genes and differential gene expression		
PD	NRXN1	animal study	adult Wistar rats were divided into five treatment groups, with 6–8 rats in each group	neurexin 1 is significantly decreased in the striatum of 6-OHDA-induced rats	[110]
			experimental groups had 6-OHDA brain injections with or without different concentrations of allopregnanolone	treatment with allopregnanolone attenuates this and other molecular changes	
			western blots were performed to evaluate the levels of the synaptic proteins PSD95 and neurexin 1 in the striatum		
PD	NRXN1	RNA expression analysis	MRI data from 149 PD patients and 64 healthy controls were obtained from the PPMI database	neurexin 1 does not have an expression pattern that predicts regional atrophy in PD	[111]
			17 genes of interest implicated in PD were selected for whole-brain expression analysis		
PD	NRXN1	animal study	adult Wistar rats were divided into seven treatment groups, with seven rats in each group	neurexin 1 expression is decreased in the striatum of 6-OHDA-induced rats	[112]
			experimental groups had 6-OHDA brain injections with or without different concentrations of apelin-13	6-OHDA rats treated with apelin-13 showed increased neurexin 1 expression in the striatum	
			western blots were performed to evaluate the levels of the synaptic proteins PSD95, neurexin 1 and neuroligin in the striatum		
spinal muscular atrophy (SMA)	NRXN2α	animal study	used HB9:D3cpv/MN-transgenic zebrafish and Smn–/−/SMN2 mice	*SMN*-deficiency downregulates neurexin 2α expression and alters its splicing in zebrafish and mouse models of SMA	[113]
			isolated total RNA from both models		
			performed a microarray analyses and qRT–PCR		

### 5.1. Human studies

Studies examining cerebrospinal fluid (CSF) from AD patients have observed lowered expression of neurexin 1 [81], as well as neurexin 2α and neurexin 3α [85]. In addition, it was found that these changes precede the neurodegeneration markers as they were observed in the preclinical stage 1 of AD [85]. Moreover, Aβ_42_ fibrils in CSF were found to bind to neurexin 1, 2 and 3 as well as proteoglycans and growth factors [83]. Levels of the synaptic proteins neuronal pentraxin 2 (NPTX2), GluA4-containing glutamate (AMPA4), neuroligin 1 and neurexin 2α are also declined in plasma neuron-derived exomes and this decline was associated with AD progression [82]. Neurexin 3 protein expression has also been seen to be specifically downregulated in blood samples of AD patients [84].

Another expression analysis on CSF from MS patients identified neurexin 2α levels as a potential biomarker for the disorder [98], while a genetic analysis found that a mutant miRNA, MIR8485, overexpresses neurexin, which leads to a calcium overload in pre-synapses [99]. It was hypothesized that this could induce neurodegeneration in MS [99].

A study examining gene expression in brain tissue samples of patients with PD found that genes related to nerve function, such as protocadherin-8 (PCDH8) and neurexin 3, were downregulated [109].

Two studies on mild cognitive impairment (MCI) found promising results. MCI is a milder form of dementia that is considered the intermediate state of cognitive decline between normal ageing and dementia [114]. Berchtold *et al.* [95] found that neurexin 1 and neurexin 2 are upregulated in MCI. In addition, neurexin 1 expression was found to be associated with longitudinal phenotypes in MCI, but not in AD [96].

One study examined neurexins in order to identify genes that are differentially regulated by HIV encephalitis [94]. This microarray study showed that neurexin 1 is downregulated in HIV encephalitis.

Finally, González-Velasco *et al.* [92] showed that neurexin 2 and neurexin 3 mRNA levels are downregulated in ageing. Another study found that neurexin 1, 2 and 3 underwent altered expression in both AD and ageing [86]. A more recent study from the same group confirmed decreased expression of both neurexin 1 and neurexin 3 in AD and ageing [88]. Interestingly, they also found that late-life physical activity is associated with increased expression of these proteins [88].

#### 5.1.1. Association study

A GWAS performed by Stein *et al.* [80] showed that the SNP rs7155434 within *NRXN3* is associated with temporal lobe structure in AD patients. Temporal lobe volume deficits are a known risk factor for AD; therefore, this study potentially implicates *NRXN3* with AD risk [80].

### 5.2. *In vitro* and *in vivo* models of disease

Several studies involving cell culture and/or rodent disease models have also shown differences in the expression of neurexin proteins. Three studies showed that neurexin 1 is downregulated in PD. One of these measured neurexin 1 mRNA in two 6-OHDA (6-hydroxydopamine)-induced cell culture models; one using human neuroblastoma (SH-SY5Y) cells and the other using primary mouse mesencephalic cells [107]. The other studies used a 6-OHDA-induced rat model of PD and both saw a decrease in neurexin 1 in the striatum [110,112]. In addition, these studies showed that treatment with apelin-13 [112] or allopregnanolone [110] is able to attenuate this change. Apelin-13 is an endogenous ligand for APJ [115] that has been investigated as a potential protective neuropeptide due to the role of the apelin-APJ system in neuronal survival [116], while allopregnanolone is a reduced metabolite of progesterone [117] and has reduced CSF levels in PD patients [118]. Freeze *et al.* [111], however, noted that the expression pattern of neurexin 1 does not predict regional atrophy in PD. This suggests that neurexin 1 is not a marker for PD; however, it does not exclude it as an important protein in PD pathogenesis. Another study in PD-transgenic mice overexpressing α-synuclein found that neurexin 2 expression was also upregulated [108]. In addition, chronic administration of cholesterol oximes was able to increase the transcription of cytoprotective genes and undo transcriptome alterations, including the alteration of neurexin 2 expression [108].

Two studies using induced models of MS implicated neurexins in this disorder. One study induced experimental autoimmune encephalomyelitis (EAE) in rats and observed downregulation of neurexin 3 [97]. This is a commonly used model that mimics certain aspects of MS. The other study used an *in vitro* model of MS, cultured human monocytes, and observed an association between ceramide-induced hypermethylation of DNA and neurexin 1 mRNA [100].

An animal study performed by Neuner *et al.* [87] showed that neurexin 3 is downregulated in AD-transgenic mice, but not in normal aged mice with impaired memory. However, Popesco *et al.* [89] found that neurexin 3 is downregulated in the cerebellum of aged mice. Another study found that levels of both neurexin 1 and neuroligin 3 are differentially expressed in cerebral cortex and hippocampus of mice and that these expression levels change during different stages of ageing [91]. They predicted that this may be responsible for the changes in synaptic plasticity observed with age [91]. Finally, a DNA methylation study by Tserel *et al.* [90] showed that CpG sites associated with *NRP1*, *NRXN2* and miR-29b-2 are hypomethylated in monocytes during ageing.

To date, only one study has examined neurexins in amyotrophic lateral sclerosis (ALS) and spinal muscular atrophy (SMA). In a cell culture model of ALS, neurexin 1 and other RNA targets of TDP-43 were dysregulated [93]. TDP-43 is a component of the cytoplasmic inclusion bodies present in ALS patients [93]. Fragments of TDP-43 are ubiquitinated, hyperphosphorylated and then accumulate in neurons and glia [119]. In zebrafish and mouse KO models of SMA, the *SMN*-deficiency downregulated neurexin 2α expression and altered its splicing [113]. SMA is associated with mutation or deletions in the *SMN* gene [120] and lack of the SMN protein causes degeneration and results in anterior horn cell dysfunction.

### 5.3. Models of induced neurodegeneration and toxicity

Several studies investigated neurexins in models of neurodegeneration or toxicity instead of studying a specific neurodegenerative disease.

Four studies examined the role of neurexins in models of induced neurodegeneration. Two of these studies hypothesized that neurexin 1 could play a role in neurodegeneration. The first study showed that a novel TDP-43 binding miRNA, miR-NID1 (miR-8485) is able to repress neurexin 1 and predicted that this could play a role in neurodegeneration [103]. Xiang *et al.* [104] found *in vitro* that excessive glycosylated acetylcholinesterase could competitively disrupt the neurexin 1β-neuroligin junctions and impair the integrity of glutamatergic synapses, which could lead to neurodegeneration. The other two studies showed that neurexin 3 is downregulated in animal models of neurodegeneration [101,102]. Suh *et al.* [102] saw that neurexin 3 was downregulated in the hippocampus of mice treated with kainic acid, an inducer of neurodegeneration, while Swanberg *et al.* [101] found that neurexin 3 is downregulated in rats with axonal damage caused by ventral root avulsion.

Two studies were conducted in animal models of neurotoxicity. One study exposed zebrafish to chronic, low levels of the neurotoxin domoic acid and saw an upregulation of neurexin 2α after two weeks [106]. The other study exposed rats to acute doses of sarin, which caused a persistent downregulation of neurexin 1β and breakdown of the blood–brain barrier [105].

In summary, multiple studies have shown changes in neurexin expression in AD, ALS, MS, PD and SMA. Many of these studies have observed downregulation of protein expression for neurexin 1, 2 and 3 in these disorders. Similarly, downregulation of neurexin 1, 2 and 3 were observed in disorders such as HIV encephalitis and MCI and in studies on ageing, in models of neuronal toxicity, and animal models of MS and ALS.

## 6. Concluding remarks

A clear link between synaptic dysfunction and neurodegenerative as well as neuropsychiatric disorders has been established in recent years. Our literature-based searches revealed several studies that have linked CNVs, deletions or expression changes in neurexins to different disorders. The evidence is most compelling for a role of neurexins in neuropsychiatric disorders, particularly in regard to the involvement of neurexin 1 in ASD and SCZ. Currently, there is comparatively less evidence for the involvement of neurexins in neurodegenerative disorders. Although there have been some studies that have suggested that neurexins may be important in these disorders, at this stage more experimental data are still needed to draw concrete conclusions. Therefore, it is apparent that more targeted studies in various disorders involving these genes as well as the proteins they encode are warranted. In terms of their broader biological and physiological functions, the neurexins function as molecular inducers, are involved in iron and protein binding, and play a role in cell-to-cell communication and response to stimuli, consequently making them critical for normal cell functioning. Furthermore, these proteins interact with various other proteins such as the neuroligins and the LRRTM proteins identified via protein interaction networks. This implicates the neurexins’ involvement in synaptic integrity and functioning making them promising candidates as disease genes for a wide range of brain pathologies.

In summary, this review serves to highlight the potential importance of the neurexin genes and proteins in human disease and recommends that more targeted studies on these genes and proteins are warranted. Furthermore, with the wealth of exomic and genomic sequences and genome-wide transcriptomic datasets now available, it has become plausible to interrogate them for their involvement in various human disorders, on a scale not previously possible. In addition, the human neurexin protein structures urgently need to be solved to understand the function and infer accurate protein–protein interactions as well as to understand the effect of mutations on the protein structure. Ultimately, improved knowledge on synapses and their individual components are necessary to develop novel therapeutic approaches for the emerging and exciting field of synaptopathies.

## Data accessibility

This article has no additional data.
